# A Novel Large-Scale Temperature Dominated Model for Predicting the End of the Growing Season

**DOI:** 10.1371/journal.pone.0167302

**Published:** 2016-11-28

**Authors:** Yang Fu, Zeyu Zheng, Haibo Shi, Rui Xiao

**Affiliations:** 1 Shenyang Institute of Automation, Chinese Academy of Sciences, Shenyang, PR China; 2 Key Laboratory of Network Control System, Chinese Academy of Sciences, Shenyang, PR China; 3 Department of Biostatistics and Epidemiology, University of Pennsylvania Perelman School of Medicine, Philadelphia, Pennsylvania, United States of America; Beijing Normal University, CHINA

## Abstract

Vegetation phenology regulates many ecosystem processes and is an indicator of the biological responses to climate change. It is important to model the timing of leaf senescence accurately, since the canopy duration and carbon assimilation are strongly determined by the timings of leaf senescence. However, the existing phenology models are unlikely to accurately predict the end of the growing season (EGS) on large scales, resulting in the misrepresentation of the seasonality and interannual variability of biosphere–atmosphere feedbacks and interactions in coupled global climate models. In this paper, we presented a novel large-scale temperature dominated model integrated with the physiological adaptation of plants to the local temperature to assess the spatial pattern and interannual variability of the EGS. Our model was validated in all temperate vegetation types over the Northern Hemisphere. The results indicated that our model showed better performance in representing the spatial and interannual variability of leaf senescence, compared with the original phenology model in the Integrated Biosphere Simulator (IBIS). Our model explained approximately 63% of the EGS variations, whereas the original model explained much lower variations (coefficient of determination R^2^ = 0.01–0.18). In addition, the differences between the EGS reproduced by our model and the MODIS EGS at 71.3% of the pixels were within 10 days. For the original model, it is only 26.1%. We also found that the temperature threshold (TcritTm) of grassland was lower than that of woody species in the same latitudinal zone.

## Introduction

Vegetation phenology plays a crucial role in regulating the exchanges of carbon, water and energy between the terrestrial ecosystems and the atmosphere[1–3]. Previous studies have revealed that the canopy duration and carbon assimilation are strongly determined by the timings of leaf senescence[4–6], which exhibits an increasingly delaying trend and has been related to a longer carbon uptake period in the context of global warming [7–9]. Therefore, it is of great significance to be able to accurately model the timing of leaf senescence, especially for determining the autumnal pattern of the net ecosystem carbon exchange[10, 11].

The current understanding of the processes of leaf senescence remains limited[12]. Several studies have suggested low temperatures[13] and short days[14, 15] to be the main factors in triggering leaf senescence in temperate deciduous trees. In accordance with the low temperature trigger hypothesis, Menzel[16] reported positive correlations between the August and September mean temperatures and the leaf senescence dates in *Fagus sylvatica* and *Quercus robur*. Regionally, water shortages have also been reported to be crucial[17, 18] and severe droughts were suggested to invoke possible factors that hasten leaf fall in deciduous species[19]. However, none of these proposed hypotheses has been thoroughly validated[20].

Consequently, there are no phenology models that can provide an accurate assessment of the end of the growing season (EGS)[21, 22]. Many models that have been integrated into various global dynamic vegetation models are merely based on empirical relationships to predict the EGS[23, 24]. For example, in the Integrated Biosphere Simulator (IBIS) model, winter-deciduous plants (temperate deciduous trees, boreal deciduous trees, cool grasses and warm grasses) drop their leaves when the daily average temperatures fall below a critical temperature threshold (5°C for deciduous trees and warm grasses and 0°C for cool grasses)[25]. Moreover, some empirical phenology models have not been validated on large scales. For example, White et al. (1997) used satellite data to calibrate a phenology model, but the calibration was only conducted at the North American but not globally[23]. Therefore, the current phenological modules are largely biased in predicting the EGS, resulting in poor performances of these dynamic vegetation modules [26].

Remote sensing data from satellites provide effective information of vegetation phenology at different scales and can be used to calibrate the phenology models[27–29]. As an example, Atkinson et al. (2012) estimated the vegetation phenological parameters in India using the satellite sensor observations[30]. Currently, remote sensing-based phenology is generally calculated from the Advanced Very High Resolution Radiometer (AVHRR), Système Pour L'Observation de la Terre (SPOT)-VEGETATION(VGT), Moderate Resolution Imaging Spectro-radiometer (MODIS) and Indian Remote Sensing (IRS)-Wide Field Sensor (WiFS) sensors[31–33]. In particular, the latest version of the MODIS Land Cover Dynamics Product (MCD12Q2) has become a commonly used phenology dataset, which provides complete and valuable phenology information on large scales for the present study[34].

Based on the global satellite-based phenological observations, the primary objectives of this study are (1) to present a novel large-scale temperature dominated phenology model for the EGS integrating with the physiological adaptation of plants to the local temperature; (2) to compare the performances of our model with the original phenology model which has been integrated into the Integrated Biosphere Simulator (IBIS); (3) to assess the spatial pattern and interannual variability of the EGS in the Northern Hemisphere using our phenology model; and (4) to calibrate the temperature threshold (TcritTm) of the EGS and exhibit the spatial pattern of the temperature threshold TcritTm from our phenology model in the Northern Hemisphere.

## Data and Methods

### 1. Satellite and meteorological data

The V005 MODIS Land Cover Dynamics (MCD12Q2) product (informally called the MODIS Global Vegetation Phenology product) was used to estimate the timing of the vegetation EGS in the study area. The MCD12Q2 product identifies the vegetation growth, maturity and senescence that mark the seasonal cycles at global scales with a spatial resolution of 500 m ×500 m and is available from 2001 to 2010[35]. This product is generated each year from the 8-day vegetation index EVI (Enhanced Vegetation Index) calculated from the NBAR reflectance (Nadir Bidirectional Reflectance Distribution Function-Adjusted Reflectance). Previous studies have provided the complete details regarding the algorithm implementation [31, 35].

**Fig 1:**
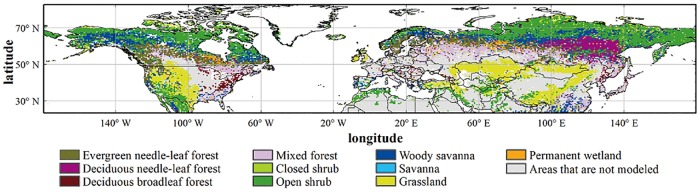
Vegetation distribution map of the Northern Hemisphere retrieved from the V005 MODIS Land Cover Type Product (MCD12Q1). Grey areas are either excluded vegetation types, such as croplands, or areas with no seasonal cycle detectable by satellite. The maps were created by the ArcMap 9.3. The data is freely provided by the Land Processes Distributed Active Archive Center (LP DAAC) (https://lpdaac.usgs.gov/data_access/data_pool.).

The V005 MODIS Land Cover Type Product (MCD12Q1) was used to identify the land cover properties. The product provides data characterizing five global land cover classification systems at annual time step and a spatial resolution of 500 m × 500 m for the period of 2001-present[36]. In this study, we chose the International Geosphere Biosphere Program (IGBP) classification scheme, which includes 11 natural vegetation classes, three developed and mosaicked land classes, and three non-vegetated land classes. We excluded the evergreen broadleaf forest from our analysis because it has little or no leaf seasonal cycles[37]. We also excluded croplands and crop/natural vegetation mosaic because human management practices strongly impact their phenology (e.g., irrigation and fertilization)[38, 39]. We validated our model and compared the modeling performances over the Northern Hemisphere (Fig 1) with the original model which was based on the low temperature trigger hypothesis[13] and has been integrated into the Integrated Biosphere Simulator (IBIS). We did not include the Southern Hemisphere and the tropical regions because of the poor performance of the MODIS Land Cover Dynamics Product over these regions[40]. Information on the datasets (MCD12Q1 and MCD12Q2) was obtained from http://lpdaac.usgs.gov, which is maintained by the NASA Land Processes Distributed Active Archive Center (LP DAAC) at the USGS/Earth Resources Observation and Science (EROS) Center, Sioux Falls, South Dakota [41, 42].

Daily temperature data was derived from the MERRA (Modern Era Retrospective-Analysis for Research and Applications) available from 2001 to 2010. MERRA is a NASA reanalysis of the data for the satellite era using a major new version of the Goddard Earth Observing System Data Assimilation System Version 5 (GEOS-5)[43]. MERRA utilizes data from all available surface weather observations globally every 3 hours, and GEOS-5 is used to interpolate and grid these point data on a short time sequence and to produce an estimate of the climatic conditions for the world at 10 meters above the land surface (approximating canopy height conditions) at a resolution of 0.5° × 0.67°[44]. Various meteorological factors (i.e., humidity, temperature, radiation, precipitation and energy balance) in the MERRA reanalysis dataset has been validated carefully at the global scale using the observed surface meteorological datasets[45, 46]. Detailed information regarding the MERRA dataset is available at the website(http://gmao.gsfc.nasa.gov/research/merra).

### 2. Our phenology modeling framework

**Fig 2:**
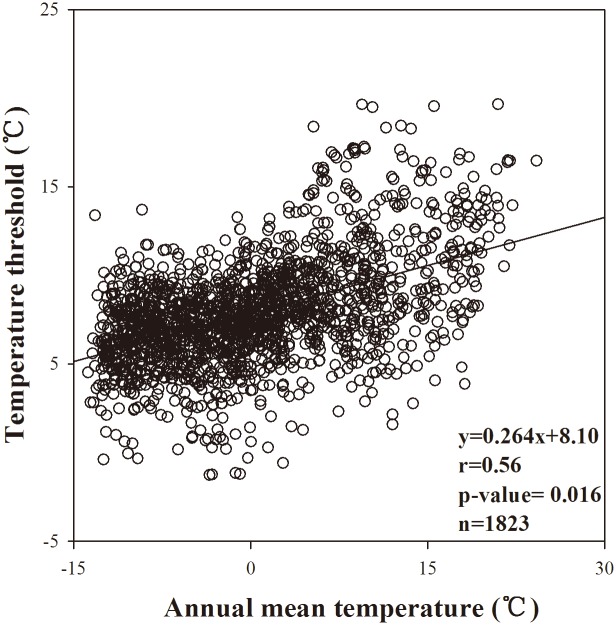
Relationship between temperature threshold for MODIS-derived EGS (end of growing season) and the average annual temperature. The solid line is fitted linear regression line.

The original phenology model which has been integrated into the Integrated Biosphere Simulator (IBIS) assumed that leaf fall for trees was initiated when one of the following conditions was met: either the average temperature (using a 10-day running average) fell below 0°C or was 5°C warmer than the coldest monthly temperature. For grasses and shrubs, leaf fall was initiated when the 10-day running average temperature reaches 0°C[25, 47]. Drought deciduous plants were assumed to drop leaves when the 10-day-mean photosynthesis rate became negative. We analyzed the relationship between average annual temperature and the 10-day running average temperature when the MODIS EGS began, and found that these two variables were linearly correlated (Fig 2). It could be explained by the physiological adaptation of plants to the local temperature environment[48]. Therefore we improved the original model by adding a regulation factor of plant adaptability *T*_*avg*_ in which the senescence began for winter-deciduous plants over the Northern Hemisphere if it was past July 1st and the10-day running average of temperatures was below a critical value (TcritTm)[23]. The critical value TcritTm was defined by:

$$\text{TcritTm }=\text{a}+\text{b}\times {\text{T}}_{\text{avg}}$$ (1)

where *T*_*avg*_ is the average annual temperature, and *a* and *b* are unknown parameters to be estimated from the data.

In each vegetation type, we used the random function of SAS to selected half of the pixels to calibrate the model parameters and validated the models using the other half of the pixels. The nonlinear regression procedure (Proc NLIN) implemented in SAS 9.2 (SAS Institute Inc., Cary, NC, USA) was applied to optimize the parameter values of the phenology model we proposed. Newton Raphson algorithm was used to train the data and the optimal model parameters in Eq (1) were obtained when the error sum of squares (ESS) was minimized. The details of the calibrated parameter values of the phenology models are shown in Table 1. For the phenology data, all dates were transformed to days of the year (DOY) for convenience of the data analysis.

**Table 1:** The calibrated parameters of our model.

Biome	Model parameters	
	a	b
Evergreen needle-leaf forest	8	0.272
Deciduous needle-leaf forest	8	0.038
Deciduous broadleaf forest	8	0.02
Mixed forest	8	0.02
Closed shrub	8	0.02
Open shrub	7	0.02
Woody savanna	8	0.02
Savanna	8	0.238
Grassland	7	0.02
Permanent wetland	8	0.02

## Results

**Fig 3:**
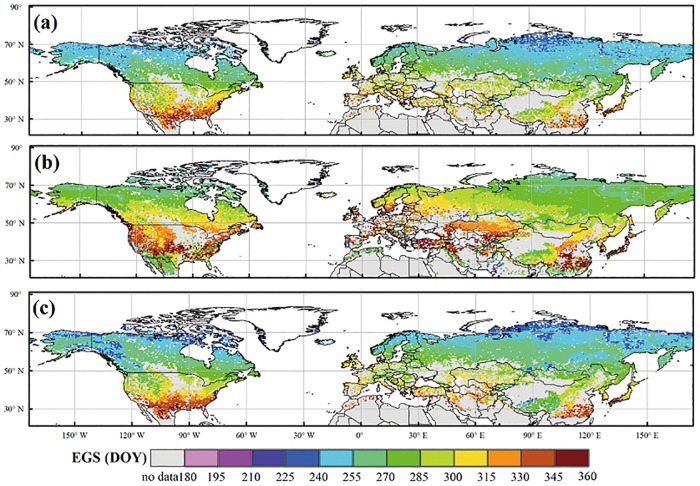
Spatial pattern of the mean dates for the EGS in the Northern Hemisphere for 2001–2010. (a) The dates derived from the MODIS product; (b) the EGS dates derived from the original model; (c) the EGS dates derived from our model. The maps were created by the ArcMap 9.3.

**Fig 4:**
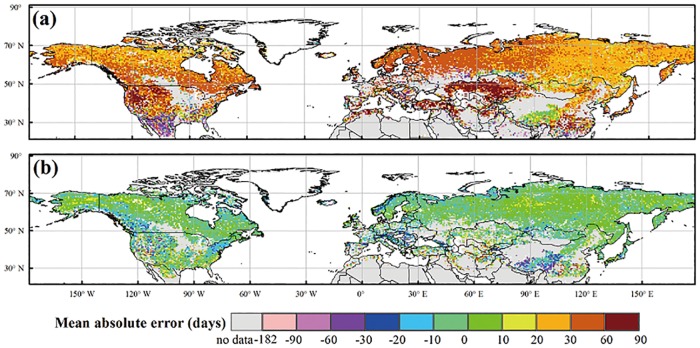
Spatial pattern of the mean absolute error (R_A_) of the EGS that simulated by the original model (a) and our phenology model in the Northern Hemisphere. The maps were created by the ArcMap 9.3.

**Fig 5:**
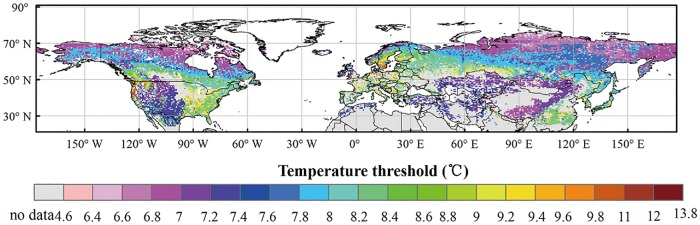
Spatial pattern of the temperature threshold (TcritTm) from our phenology model in the Northern Hemisphere. The maps were created by the ArcMap 9.3.

The EGS simulated by our model are better agreed to the satellite-derived EGS, compared to those calculated by the original phenology model in IBIS (Fig 3). An early EGS was found in the boreal and cool regions, intermediate EGS in the temperate regions and late EGS in the warm regions. In terms of the spatial patterns of the mean absolute error (R_A_), our model outperformed the original model (Fig 4). The results indicated lower R_A_ of our simulations in most of the boreal and cool regions, for which the R_A_ was less than 10 days (Fig 4b). In contrast, the results of the original model delayed the timing of the EGS by 10–30 days compared with the MODIS EGS in the boreal and cool regions and predicted an earlier EGS of 30–90 days compared with the MODIS EGS in the woody savanna and open shrub areas of low latitudes (Fig 4a). Furthermore, the calibrated temperature threshold (TcritTm) in our phenology model exhibited obvious spatial variations in the Northern Hemisphere (Fig 5). The result indicated that the temperature threshold was approximately 7–9°C and exhibited an increasing trend from north to south in the Northern Hemisphere. In the same latitudinal zone, the temperature threshold (TcritTm) of grassland was lower than that of woody species.

**Fig 6:**
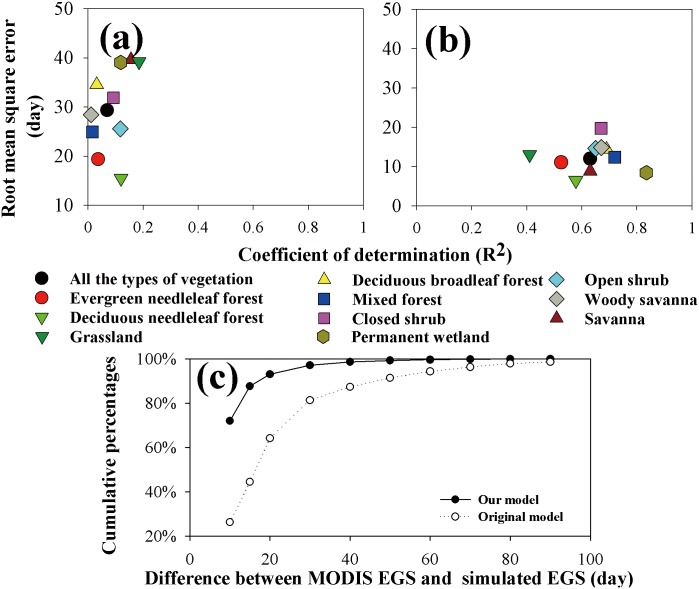
Coefficient of determination (R^2^) and root mean square error (RMSE) for (a) the original and (b) our phenology models for the various vegetation types over the Northern Hemisphere, and(c) cumulative percentage of the absolute differences between the MODIS EGS and the simulated EGS from the original and our phenology models.

Statistically, our model explained approximately 63% of the EGS variations over the Northern Hemisphere (Fig 6b). The coefficients of determination (R^2^) varied from 0.41 (grassland) to 0.83 (permanent wetland). The average root mean square error (RMSE) varied from 6 days (deciduous needle-leaf forest) to 19 days (closed shrub). In contrast, the original model had a low R^2^ (0.01–0.18), and the average RMSE ranged from 15 to 47 days (Fig 6a). The cumulated frequencies of the absolute difference between the EGS simulations and the MODIS EGS further demonstrated that our model produced significantly improved results compared to the original model (Fig 6c). Overall, our model reproduced the timing of the EGS for 71.3% of the pixels within 10 days of the MODIS EGS and for 87.8% within 15 days. In contrast, the original model reproduced the timing of the EGS for 26.1% of the pixels within 10 days of the MODIS EGS and for 45.0% within 15 days.

**Fig 7:**
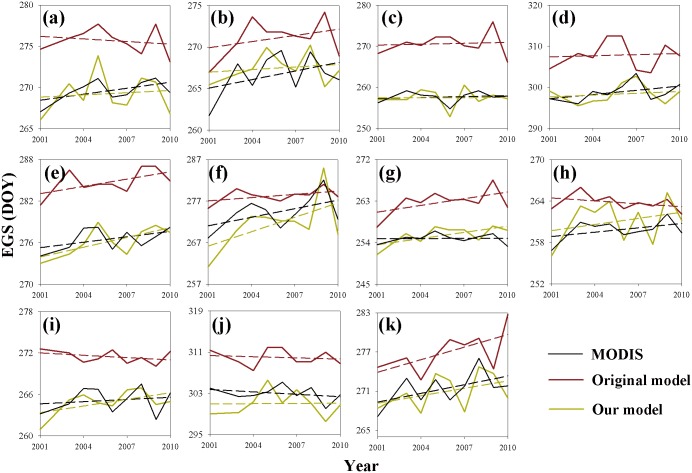
Interannual variability of the end dates of the growing season from the MODIS product and the phenology models for the period of 2001 to 2010. (a) All of the types of vegetation; (b) evergreen needle-leaf forest; (c) deciduous needle-leaf forest; (d) deciduous broadleaf forest; (e) mixed forest; (f) closed shrub; (g) open shrub; (h) woody savanna; (i) savanna; (j) grassland; (k) permanent wetland. The short dashed lines are regression lines.

The magnitude and long-term change trends of the EGS simulated by our model were significantly different from that calculated by the original phenology model in IBIS (Fig 7). The dates derived from our model were consistent with those of the MODIS EGS, whereas the dates derived by the original model were greatly delayed. For example, from 2001 to 2010, the average EGS simulated by our and original phenology models were, respectively, the 297^th^ and 309^th^ day in the deciduous broadleaf forest region, compared with the 298^th^ day from the MODIS EGS (Fig 7d). Moreover, the original model exhibited large differences in terms of the interannual variability of the EGS. For example, our model and the MODIS EGS both showed a significantly increasing trend from 2001 to 2010, while the original model indicated significantly decreasing trends in the woody savanna area (Fig 7h).

## Discussion

Vegetation phenology serves a crucial function in regulating many ecosystem processes and is a key indicator of the biological responses to climate change[49]. Predicting the impact of changing phenology on terrestrial ecosystems requires an accurate phenology model[50]. In this study, we presented a novel large-scale temperature dominated phenology model and showed that this model provided more accurate prediction of EGS compared to the original phenology model. Our phenology model outperforms the original model by using the mean annual temperature to determine the minimum temperature threshold. Vegetation phenology is the optimization of the plant activity and reproduction resulting from natural selection[51]. Plant species have adapted their temperature requirements to their local temperature environment[52–54]. Thus it is essential to integrate the physiological adaptation of plants to the local temperature into the phenology models and improve model performance at the global scale.

This study calibrated the temperature threshold (TcritTm) and derived the spatial pattern of the TcritTm from our phenology model in the Northern Hemisphere. The result indicated that the temperature threshold was approximately 7–9°C, which is consistent to a previous study, which suggested that a threshold of 8°C is a main factor in triggering leaf senescence for four deciduous tree species (horse chestnut, beech, birch and oak) in Germany[13]. The spatial pattern of the TcritTm suggested an increasing trend from north to south in the Northern Hemisphere. A low temperature threshold was found in the boreal and cool regions, an intermediate temperature threshold in the temperate regions and a high temperature threshold in the warm regions. This trend is consistent with the change trend of temperature from the south to the north, reflecting the adaptability of plants to the local temperature environment[55, 56]. Moreover, it should be noted that the temperature threshold (TcritTm) of grassland was lower than that of woody species in the same latitudinal zone. This phenomenon might be related to the local climate and plant species[57].

The photoperiod and temperature have frequently been reported as the main drivers of leaf senescence[58, 59]. However, when tested over extensive datasets, the temperature, which is considered as an independent variable in phenology models, appeared to explain a higher proportion of the observed variance in the timing of senescence[60, 61]. Our phenology model based on the minimum temperature threshold explained most of the variability of leaf senescence in the Northern Hemisphere, which agreed with previous reports that claimed temperature plays the primary role in determining leaf coloring for *Quercus*[20]. Thus, it may be feasible to solely consider the influence of temperature on the timing of the EGS in plant phenology models.

Our study was based on the V005 MODIS Land Cover Dynamics (MCD12Q2) product, and there was some uncertainty exist in the product[62, 63]. For example, Ganguly et al. (2010) compared the MODIS EGS with field measurements of forest canopy phenology at Harvard Forest for 2001–2006 and found that the average date of the EGS slightly differ from the MODIS EGS [35]. The uncertainty from this product may affect the simulation of the phenology model. In addition, the MODIS EGS exhibited large uncertainties in the tropics[40]. Therefore, we did not calibrate and examined our phenology model in the tropics for the present study. Overall, further efforts focusing on increasing the precision of the phenology products are needed to improve phenology models.

## Summary

This study presented a novel large-scale temperature dominated model for predicting the end of the growing season and compared the performances of our model with the original phenology model which has been integrated into the Integrated Biosphere Simulator (IBIS). The results indicated that the novel large-scale temperature dominated phenology model explained most of the EGS variations over the Northern Hemisphere and greatly improves the accuracy compared with the original model. When spatially averaged, predictions of our phenology model exhibited very good agreement with mean annual dates of leaf senescence. We consider the novel large-scale temperature dominated model to be a primary tool for predicting leaf senescence.

## Supporting Information

S1 File The original data of MCD12Q2 product from 2001 to 2010. (ZIP)

S2 File The original data of MCD12Q1 product. (ZIP)
